# Arylsulfatase D promotes malignant phenotypes in glioblastoma cells and is linked to altered Hippo pathway phosphorylation

**DOI:** 10.3389/fonc.2026.1927290

**Published:** 2026-08-24

**Authors:** Xuzhao Li, Qifan Hou, Yanli Tang, Nianhua Wang, Chen Zhong, Lixin Xu, Jun Wang

**Affiliations:** Department of Neurosurgery, Changde Hospital, Xiangya School of Medicine, Central South University (The First People’s Hospital of Changde City), Changde, Hunan, China

**Keywords:** arylsulfatase D, glioblastoma, hippo pathway, irosustat, malignant phenotype, matrigel-based tube-like network formation, YAP phosphorylation

## Abstract

**Introduction:**

Glioblastoma (GBM) is a highly aggressive malignant brain tumor with persistently poor clinical outcomes and few effective treatment options. Arylsulfatase D (ARSD), a lysosomal sulfatase implicated in several cancers, has not been fully characterized in GBM.

**Methods:**

ARSD expression in GBM was evaluated using bioinformatic analyses and immunohistochemical validation. ARSD was overexpressed or knocked down in U87MG, U251MG, and A172 cells, and proliferation, three-dimensional spheroid growth, apoptosis, invasion, and Matrigel-based tube-like network formation were assessed. Merlin/LATS/YAP phosphorylation-related protein patterns were analyzed. Irosustat was further evaluated in a subcutaneous U87MG xenograft model.

**Results:**

ARSD was upregulated in GBM tissues, and higher ARSD expression was associated with unfavorable patient survival. ARSD overexpression enhanced proliferation, three-dimensional spheroid growth, invasion, and Matrigel-based tube-like network formation while reducing apoptosis, whereas ARSD knockdown suppressed these malignant phenotypes. ARSD expression was associated with altered Merlin/LATS/YAP phosphorylation-related protein patterns, particularly changes in YAP1 abundance and phosphorylation status. In the xenograft model, Irosustat treatment altered YAP phosphorylation-associated protein patterns, enhanced tumor cell apoptosis, reduced tumor burden, and prolonged mouse survival.

**Discussion:**

These findings indicate that ARSD facilitates GBM malignant behavior and is linked to Merlin/LATS/YAP phosphorylation-related signaling. Irosustat showed preliminary antitumor activity in the xenograft model; however, because Irosustat is not an ARSD-specific inhibitor, the in vivo findings should not be interpreted as evidence of selective ARSD inhibition.

## 1 Introduction

GBM represents the most malignant form of adult-type diffuse glioma and is among the deadliest primary tumors of the central nervous system. According to the 2021 World Health Organization classification of central nervous system tumors, GBM is defined through integrated histological and molecular criteria as an IDH-wildtype diffuse astrocytic tumor with grade 4 biological behavior (1, 2). The U87MG, U251MG, and A172 cell lines used in the present study are established GBM-derived experimental models and should not be regarded as molecularly classified patient tumors under the current integrated WHO diagnostic framework.Despite progress in molecular classification and clinical management, the prognosis of GBM remains poor. Standard management generally consists of maximal safe resection followed by radiotherapy and TMZ-based chemotherapy, as established by the Stupp regimen and subsequent long-term follow-up studies (3, 4). However, diffuse infiltration, therapeutic resistance, intratumoral heterogeneity, and recurrence continue to limit durable clinical benefit (5, 6). Recent single-cell and molecular pathology studies have further highlighted the complex cellular and genetic heterogeneity of GBM, reinforcing the need to identify functional regulators that contribute to malignant phenotypes and treatment resistance (7, 8).

The Hippo pathway is a conserved signaling network involved in tissue homeostasis, organ growth, cell proliferation, apoptosis, and tumor development (9). YAP and TAZ function as major downstream transcriptional coactivators within this pathway and are increasingly viewed as central mediators of cancer cell plasticity, proliferation, invasion, and therapy resistance (10). In the canonical Hippo kinase cascade, upstream components including NF2/Merlin and LATS1/2 promote inhibitory phosphorylation of YAP/TAZ, thereby restricting their nuclear accumulation and transcriptional activity. Conversely, reduced inhibitory phosphorylation of YAP/TAZ can facilitate their interaction with TEAD transcription factors and promote gene programs related to cell growth, survival, migration, extracellular matrix remodeling, and malignant progression (9–11). In glioma, YAP-associated signaling has been reported to promote tumor growth through crosstalk with Wnt/β-catenin signaling and autophagy-related mechanisms (12, 13). In addition, pharmacological interference with the YAP–TEAD axis has shown anti-invasive and anti-proliferative effects in preclinical GBM models (14). These findings suggest that altered Hippo/YAP-axis signaling is relevant to GBM biology, although the upstream molecules that modulate Merlin/LATS/YAP phosphorylation-related protein patterns in GBM remain incompletely characterized.

In addition to proliferation and invasion, GBM cells may acquire vascular-like phenotypes that contribute to tumor adaptation and aggressiveness. Vasculogenic mimicry-like tube formation has been reported in glioblastoma and glioma models and is considered distinct from classical endothelial-cell-driven angiogenesis (15, 16). Therefore, evaluating tube-like network formation in GBM cells may provide complementary information regarding malignant plasticity, provided that such findings are interpreted as vasculogenic mimicry-like phenotypes rather than definitive angiogenesis. Identifying molecular regulators that coordinate proliferation, invasion, apoptosis resistance, spheroid growth, and tube-like formation may help clarify the biological programs underlying GBM progression.

Arylsulfatase D (ARSD) is a lysosomal sulfatase belonging to the arylsulfatase family. Recent evidence has suggested that ARSD may have cancer-related functions. In glioma, ARSD was reported to serve as a prognostic biomarker and promote tumor progression through the JAK2/STAT3 pathway and macrophage-associated immune infiltration (17). In breast cancer, ARSD was described as an ERα downstream target and was shown to restrain proliferation and migration of triple-negative breast cancer cells by activating Hippo/YAP signaling (18). These findings suggest that the biological function of ARSD may be tumor-context dependent. However, the expression pattern and functional relevance of ARSD in GBM, as well as its association with Merlin/LATS/YAP phosphorylation-related signaling, have not been fully defined.

Steroid sulfatase and related sulfatase biology have also attracted attention as pharmacologically actionable processes in cancer. Irosustat, also termed STX64 or 667-Coumate, is an orally available first-generation irreversible steroid sulfatase inhibitor that has been tested in xenograft models and clinical studies (19, 20). In previous *in vivo* xenograft studies, oral STX64/Irosustat at 10 mg/kg daily showed tumor growth-inhibitory activity and effective suppression of steroid sulfatase activity, providing a pharmacological basis for *in vivo* dosing. Although Irosustat is not an ARSD-specific inhibitor, it provides a known sulfatase inhibitor for exploring whether pharmacological sulfatase inhibition may influence GBM xenograft growth. In the present study, we investigated ARSD expression in GBM using public datasets and clinical tissue validation, examined the effects of ARSD gain- and loss-of-function on GBM cell proliferation, spheroid growth, apoptosis, invasion, and Matrigel-based tube-like network formation, and analyzed associated changes in Merlin/LATS/YAP phosphorylation-related protein patterns. We further evaluated Irosustat treatment in a subcutaneous U87MG xenograft model to explore the preliminary anti-tumor activity of pharmacological sulfatase inhibition *in vivo*.

## 2 Materials and methods

### 2.1 Bioinformatics analysis

The bulk expression profile of ARSD across normal human tissues was obtained from the GTEx Portal using the ARSD gene entry (ENSG00000006756.16), based on GTEx Analysis Release V10 (21). Pan-cancer ARSD expression across tumor and paired normal samples was analyzed using GEPIA, an online platform integrating TCGA tumor data and GTEx normal tissue data (22). Differential ARSD expression between GBM and non-tumor brain tissues was also evaluated using GEPIA according to the instructions of the database. Overall survival analysis according to ARSD expression was performed using GEPIA, and patients were stratified into high- and low-expression groups according to the median ARSD expression level. Single-cell analysis of ARSD expression in GBM was performed using the TISCH database (23). ARSD distribution across malignant and non-malignant cell populations was examined using the GBM single-cell datasets GSE103224 and GSE102130 available in TISCH (23). The TISCH datasets were used descriptively to visualize ARSD expression distributions across annotated cell populations; no formal statistical enrichment analysis was performed.

### 2.2 Immunohistochemical staining

Archived paraffin-embedded GBM samples were collected from 35 patients treated at the First People’s Hospital of Changde City between June 2017 and July 2019, including 20 men and 15 women. The included patients had a median age of 51 years, with an age range of 37–65 years. Among these archived specimens, three paired GBM tissues and matched non-tumor peritumoral tissues with adequate tissue quality and complete paired sampling were selected for immunohistochemical validation. The matched comparator samples were designated as non-tumor peritumoral tissues rather than normal brain tissue. The absence of obvious tumor cell infiltration was confirmed by histopathological examination of hematoxylin and eosin-stained sections by two experienced pathologists. Tissue sections were incubated with an anti-ARSD primary antibody (1:200; Invitrogen, USA) and detected using a DAB substrate kit (Cell Signaling Technology, 8059P, USA). Immunohistochemistry (IHC) staining was evaluated according to staining intensity as previously described (24). Scores were assigned as 0 for negative staining, 1 for weak staining, 2 for moderate staining, and 3 for strong staining. For each specimen, three randomly chosen microscopic fields were scored, and the mean value was used as one biological replicate. Written informed consent was obtained from all patients. The study was approved by the Ethics Committee of the First People’s Hospital of Changde City (2024–087–01).

### 2.3 ARSD overexpression and knockdown in GBM cell lines

U87MG, U251MG, and A172 cells were obtained from Pronosay (Wuhan, China) and used at low passage numbers after recovery. ARSD overexpression was achieved using a pLenti-CMV-EGFP-based lentiviral construct carrying the human ARSD coding sequence, with the corresponding empty vector used as the control. ARSD knockdown was achieved using an ARSD-targeting shRNA lentiviral construct, with a non-targeting shRNA construct used as the control. Lentiviral particles were packaged in 293T cells, and the collected viral supernatants were used to transduce the GBM cells. Following transduction, cells were selected with 2 μg/mL puromycin until stable ARSD-overexpressing, ARSD-knockdown, and corresponding control populations were established. ARSD modulation was confirmed by quantitative real-time PCR and Western blotting before downstream experiments.

### 2.4 RNA isolation and quantitative real-time PCR

GBM cells were lysed with TRIzol reagent (Ambion, USA) to isolate total RNA following the supplier’s protocol. Complementary DNA was prepared with the PrimeScript RT kit (Takara, RR037Q, Japan). Quantitative real-time PCR was carried out using SYBR qPCR SuperMix Plus (Novo Protein, E096, China) on a ViiA 7 real-time PCR system (Applied Biosystems, USA). GAPDH served as the internal reference, and relative ARSD mRNA levels were determined by the 2−ΔΔCt method. The primer sequences were listed as follows:

ARSD forward: 5′-TCCCGCCGCCGAACACCTA-3′;

ARSD reverse: 5′-GGATCCGGCCGACCAGAGA-3′;

GAPDH forward: 5′-GTCTCCTCTGACTTCAACAG-3′;

GAPDH reverse: 5′-ACCACCCTGTTGCTGTAGCC-3′;

### 2.5 Western blot

Cells were lysed in ice-cold RIPA buffer (Beyotime, China) containing protease and phosphatase inhibitors. Equal protein quantities were resolved by SDS-PAGE and then transferred to PVDF membranes. After blocking, membranes were incubated with primary antibodies against ARSD (Affinity Biosciences, Australia), Merlin (Cell Signaling Technology, USA), LATS1, p-LATS1, LATS2, YAP1, p-YAP1, GAPDH (Proteintech, China), and p-LATS2 (Thermo Fisher Scientific, USA). HRP-linked secondary antibodies were supplied by Cell Signaling Technology, USA. Signals were visualized using enhanced chemiluminescence reagent (Thermo Fisher Scientific, USA) and captured with a Bio-Rad gel imaging system. Band intensities were quantified with ImageJ software. Total proteins were normalized to GAPDH, whereas phosphorylated LATS1, phosphorylated LATS2, and phosphorylated YAP1 were normalized to their corresponding total protein levels. Uncropped blots were provided in the **Supplementary Materials**.

### 2.6 Cell apoptosis assay

Stable ARSD-overexpressing, ARSD-knockdown, and corresponding control GBM cells were collected and labeled with FITC-conjugated Annexin V and propidium iodide with FITC-conjugated Annexin V and propidium iodide (PI) (Livning, China) following the manufacturer’s protocol. Flow cytometric detection was conducted on a BD FACSVerse instrument (BD Biosciences, USA), and FlowJo version 10.0 was used for analysis. Early and late apoptotic fractions were combined to calculate the total apoptotic cell percentage.

### 2.7 Three-dimensional spheroid culture

For *in vitro* spheroid growth assessment, U87MG, U251MG, and A172 cells were plated in ultra-low-attachment U-bottom 96-well plates at 1 × 10³ cells per well. Cells were maintained in DMEM/F-12 medium containing 1× B-27, 5 μg/mL heparin, 2 mM L-glutamine, 20 ng/mL EGF, 20 ng/mL bFGF, and 1% penicillin/streptomycin, as described previously (25, 26). After 2–3 days of culture at 37 °C, spheroids were imaged using an inverted microscope (Leica, Germany). Image analysis software was used to measure spheroid size. For each spheroid, the longest and shortest diameters were recorded, and volume was estimated as length × width²/2.

### 2.8 Cell proliferation assay

Use the cell counting kit-8 (CCK-8; BOSTER, USA) to detect cell proliferation according to the instructions of the kit. Stable ARSD-overexpressing, ARSD-knockdown, and corresponding control GBM cells were seeded in 96-well plates at a density of 2 × 10³ cells per well. From day 0 to day 4, add 10 μL CCK-8 solution at each specified time and incubate at 37 °C for 2 hours. Use an enzyme labeler to record the absorbance value at 450 nm. All experiments set up at least three biological repetitions.

### 2.9 Transwell invasion assay

Use the Transwell chamber (Falcon, United States) with aperture of 8 μm Matrigel envelope to evaluate the ability of cell invasion. Suspend U87MG, U251MG and A172 cells in serum-free culture medium and inoculate them in the upper chamber with a density of 1 × 10³ cells per hole. Add a complete culture medium containing 10% fetal bovine serum as a chemokine in the lower chamber. After 72 hours, gently wipe off the uninvaded cells on the surface of the superior ventricular membrane with a cotton swab. The cells that migrate to the surface of the inferior ventricular membrane are fixed with methanol, crystal violet staining, imaging under the microscope, and randomly selecting the field of view for counting.

### 2.10 Matrigel-based tube-like network formation assay

To evaluate the capacity of GBM cells to form short-term tube-like networks on Matrigel, 96-well plates were precoated with Matrigel (100 μL per well; BD Biosciences, USA) for 30 min at 37 °C. GBM cells were then seeded onto the Matrigel-coated plates at 2.5 × 10^4 cells per well. After 6 h of incubation, tube-like networks were photographed microscopically and quantified using Image-Pro Plus 6 software. Branch-point number was used as the quantitative indicator of tube-like network formation. Because this short-term assay assessed network formation on Matrigel without endothelial-marker validation or *in vivo* perfusion analysis, the observed structures were interpreted as tube-like networks rather than definitive evidence of bona fide vasculogenic mimicry.

### 2.11 Irosustat and temozolomide

Irosustat (STX64/667-Coumate), a known irreversible steroid sulfatase inhibitor, was purchased from MedChemExpress (HY-14586, USA). Temozolomide (TMZ) was purchased from MedChemExpress (HY-17364, USA). For *in vivo* administration, Irosustat was freshly prepared in a vehicle containing 10% tetrahydrofuran and 90% propylene glycol for oral gavage. TMZ was freshly prepared in 0.5% carboxymethylcellulose sodium solution for oral administration. Vehicle-matched control administration was performed according to the corresponding treatment schedule. The *in vivo* Irosustat dose was selected based on previous xenograft studies using oral STX64/Irosustat at 10 mg/kg daily (19).

### 2.12 Mouse xenograft model and *in vivo* treatment

Six-week-old male BALB/c nude mice were obtained from Hangzhou Ziyuan Laboratory Animal Technology, China. Only male mice were included to maintain a uniform experimental cohort in this exploratory study; consequently, sex-related differences were not assessed. Each animal received a subcutaneous injection of 5 × 10^6 U87MG cells. When tumors reached approximately 100 mm^3, mice were randomly allocated into five groups with six animals per group: model group receiving vehicle; Irosustat group receiving Irosustat orally at 10 mg/kg daily; radiotherapy (RT) group exposed to γ-rays at 2 Gy twice weekly for a total of four fractions; temozolomide (TMZ) group receiving TMZ orally at 50 mg/kg daily; and combination group receiving Irosustat, RT, and TMZ. On irradiation days, Irosustat and/or TMZ were administered 2 h before RT in the corresponding treatment groups. The RT and TMZ schedules were selected as preclinical treatment regimens for this xenograft experiment and were not intended to reproduce or be considered equivalent to the clinical Stupp protocol. Treatments and monitoring were continued for 7 weeks. Body weight was recorded every three days from day 0 to day 48 to monitor general condition and tolerability. After the study, the mice were killed and the tumor tissue was collected for follow-up pathology and molecular analysis. Animal procedures were approved by the Ethics Committee of Xiangya Hospital of Central South University (Approval No. 202408047).

### 2.13 Staining of mouse tumor sections

Mouse tumor tissues were fixed with 4% paraformaldehyde and processed for paraffin embedding. Paraffin sections were stained with a commercial hematoxylin and eosin (H&E) kit (Beyotime, China) and photographed microscopically. TUNEL staining was performed according to the manufacturer's instructions, and images were acquired at 200× magnification. For immunofluorescence analysis, serial tumor sections were incubated separately with anti-CD86 (1:50; BD Pharmingen, USA) or anti-CD68 (1:50; BD Pharmingen, USA), followed by a Cy3-conjugated secondary antibody (1:200; Jackson ImmunoResearch, UK) and DAPI counterstaining. Images were obtained at 200× magnification. For each mouse, one representative tumor section and three randomly selected non-overlapping microscopic fields were analyzed. TUNEL positivity was quantified as the percentage of TUNEL-positive nuclei among total nuclei, whereas CD68 and CD86 staining was quantified as the percentage of positively stained area using ImageJ under identical analysis settings. The three field-level measurements were averaged to obtain one value for each mouse, and the mouse, rather than the individual field, was treated as the experimental unit. Three mice per group were included in these analyses.

### 2.14 ELISA assay

After 7 weeks of treatment, blood was collected from the orbital sinus of each mouse, and plasma was separated by centrifugation. Mouse IL-6 (Invitrogen, BMS603-2; assay range, 31.3–2,000 pg/mL), IL-10 (Invitrogen, BMS614; assay range, 39–2,500 pg/mL), TNF-α (Invitrogen, BMS607-3; assay range, 31.3–2,000 pg/mL), and IFN-γ (Invitrogen, KMC4021; assay range, 47–300 pg/mL) were measured according to the manufacturers’ instructions. Plasma samples were analyzed in technical duplicate, and the mean of the duplicate measurements was used for analysis. Samples exceeding the upper assay range were remeasured after appropriate dilution. Each plotted value represents one individual mouse.

### 2.15 Statistical analysis

GraphPad Prism version (GraphPad Software, Boston, MA, USA) was used for all statistical analyses.Results are expressed as mean ± standard deviation (SD). Two independent groups were compared with an unpaired two-tailed Student’s t-test, while paired tissue data were evaluated with a paired two-tailed Student’s t-test. For datasets containing more than two groups, one-way ANOVA with Dunnett’s *post hoc* test was applied to compare each treatment group with its corresponding control. Repeated-measures datasets, including CCK-8 proliferation curves and mouse body-weight curves, were assessed by two-way repeated-measures ANOVA. Kaplan–Meier survival data were evaluated using the log-rank test. Biological replicate numbers, animal numbers, or patient specimen numbers are provided in the relevant figure legends. Statistical significance was defined as P < 0.05. Error bars represent the mean ± SD. The statistical test and sample size are stated in each figure legend, and exact P values are reported where available.

## 3 Results

### 3.1 ARSD is upregulated in GBM and associated with unfavorable prognosis

**Figure 1:**
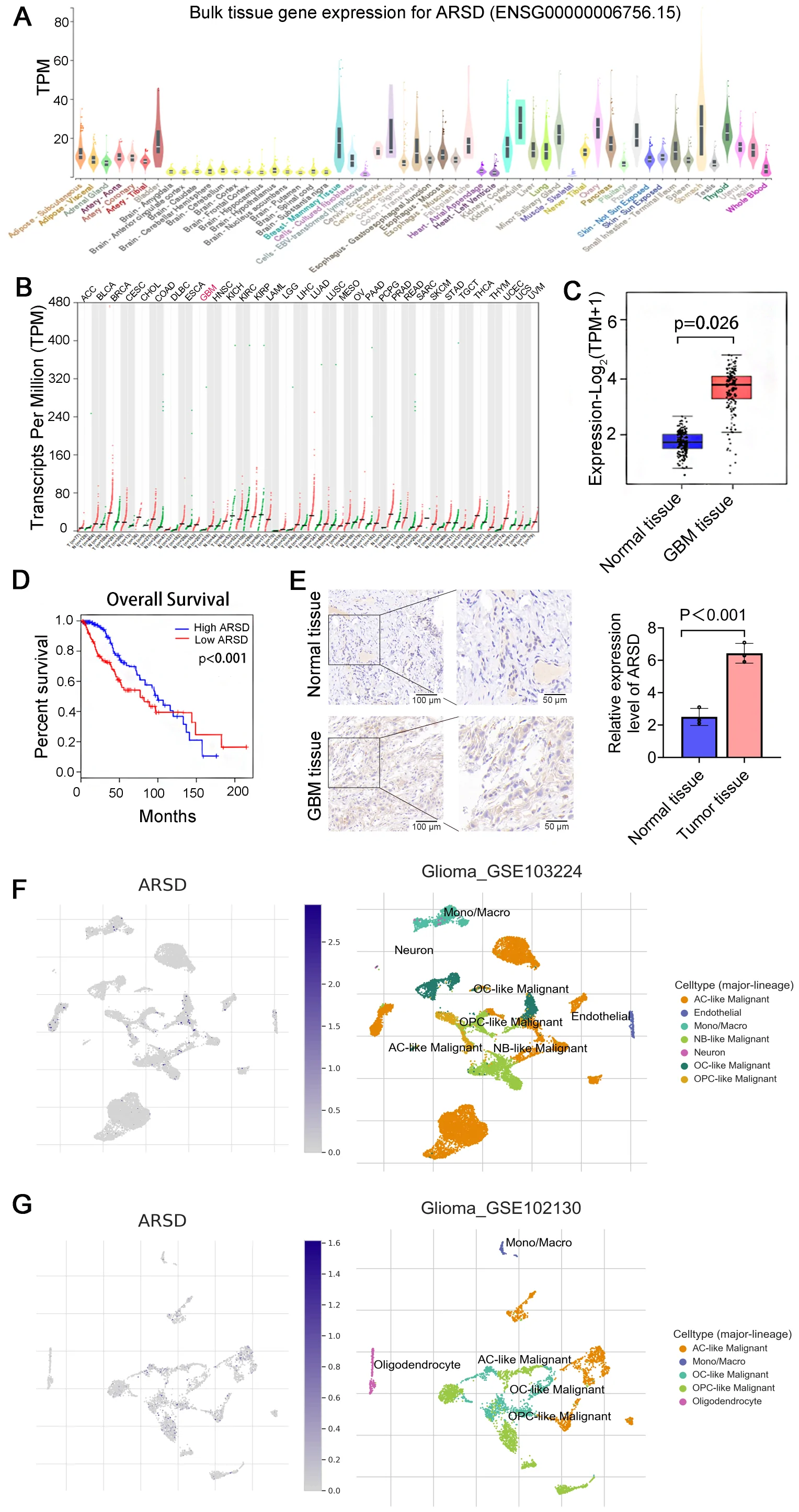
ARSD is upregulated in GBM and associated with unfavorable prognosis. **(A)** Bulk tissue expression profile of ARSD across normal human tissues obtained from the GTEx Portal. **(B)** Pan-cancer expression profile of ARSD across tumor and paired normal samples analyzed using GEPIA. **(C)** ARSD expression in non-tumor brain tissues and GBM tissues analyzed using GEPIA. Normal, n = 207; GBM, n = 163. **(D)** Overall survival analysis of GBM patients stratified by high or low ARSD expression. n = 257. **(E)** Representative immunohistochemical staining and quantification of ARSD expression in GBM tissues and matched non-tumor peritumoral tissues. Quantification was performed using three paired tissue specimens, with three randomly selected fields averaged for each specimen. Scale bars: 100 μm and 50 μm. **(F)** Single-cell analysis of ARSD expression and cell-type distribution in the GBM dataset GSE103224. **(G)** Single-cell analysis of ARSD expression and cell-type distribution in the GBM dataset GSE102130. Data are presented as mean ± SD where applicable.

We initially analyzed ARSD expression across normal human tissues using GTEx-derived bulk expression data. As shown in **Figure 1A**, ARSD expression was relatively low in matched non-tumor peritumoral tissues compared with several other normal tissue types. We next analyzed ARSD expression across multiple cancer types using GEPIA. This pan-cancer analysis revealed variable ARSD expression differences between tumor and paired normal samples among cancer types (**Figure 1B**). GBM-focused analysis further showed significantly higher ARSD expression in GBM tissues than in non-tumor brain tissues (**Figure 1C**). Survival analysis indicated that patients with high ARSD expression had shorter overall survival than those with low expression (**Figure 1D**). To further validate ARSD expression at the tissue level, immunohistochemical staining was performed using GBM tissues and matched non-tumor peritumoral tissues. Consistent with the bioinformatic findings, ARSD staining was stronger in GBM tissues than in matched non-tumor peritumoral tissues (**Figure 1E**).

We then explored the cellular distribution of ARSD expression in GBM using single-cell datasets from the TISCH database. In the GSE103224 dataset, ARSD expression was detectable in malignant cell populations, including AC-like and OPC-like malignant cells, and was also observed in Mono/Macro cell populations (**Figure 1F**). Similar cell type-associated ARSD expression patterns were observed in the GSE102130 dataset (**Figure 1G**). These descriptive single-cell visualizations indicate that ARSD expression was detectable and relatively prominent in selected malignant and non-malignant cell populations. No formal statistical enrichment analysis was performed. Together, these findings indicate that ARSD is upregulated in GBM and that higher ARSD expression is associated with unfavorable patient prognosis.

### 3.2 Genetic modulation of ARSD alters GBM cell proliferation, spheroid growth, and apoptosis

**Figure 2:**
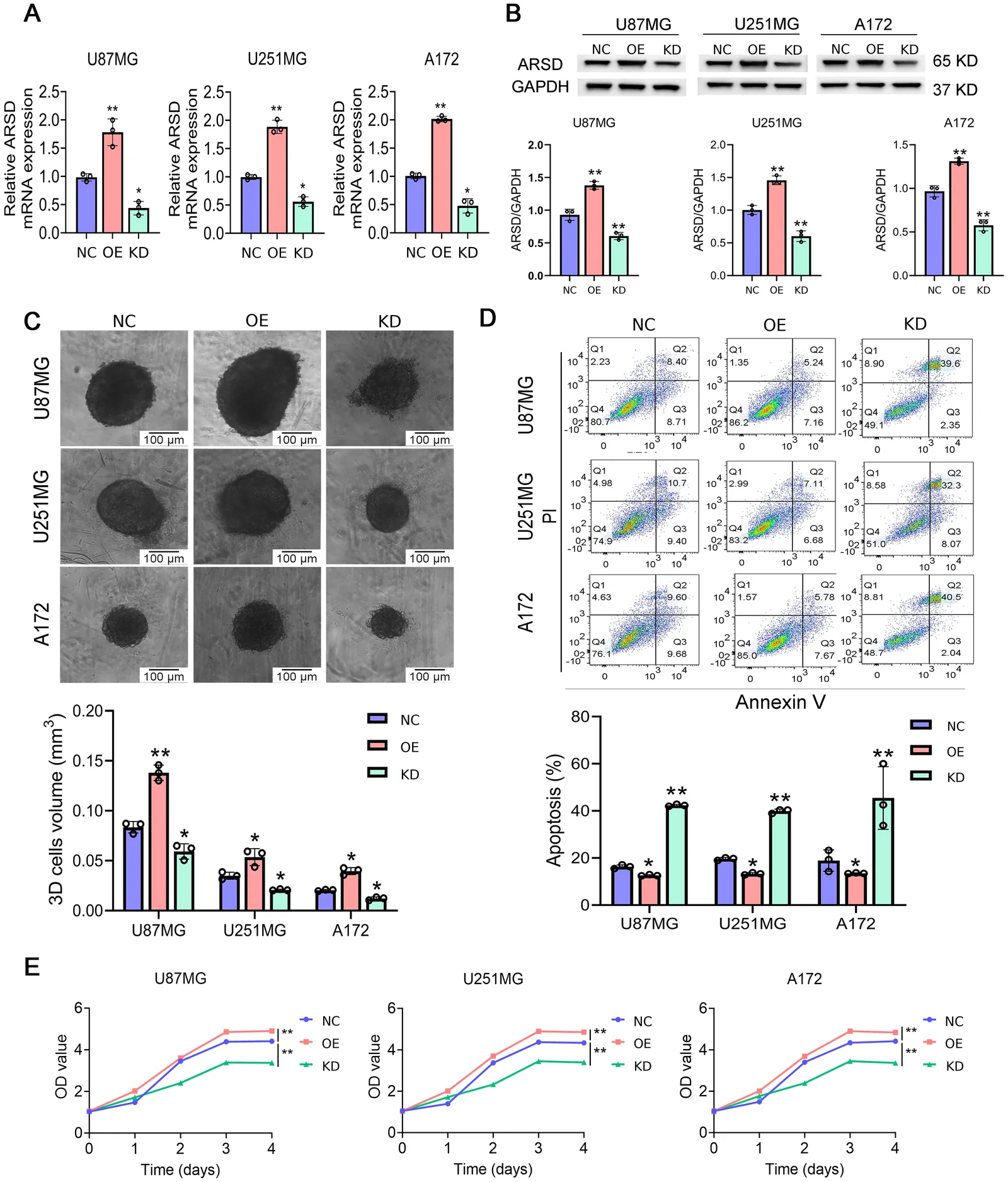
Genetic modulation of ARSD alters GBM cell proliferation, spheroid growth, and apoptosis.ARSD overexpression (OE) and knockdown (KD) were performed in U87MG, U251MG, and A172 cells. **(A)** Quantitative real-time PCR analysis showing ARSD mRNA expression after ARSD overexpression or knockdown. **(B)** Western blot analysis and quantification of ARSD protein expression after ARSD overexpression or knockdown. **(C)** Representative images and quantification of three-dimensional spheroid growth in GBM cells. Scale bars: 100 μm. **(D)** Flow cytometric analysis of apoptosis using Annexin V/PI staining and quantification of total apoptotic cells. **(E)** CCK-8 assay showing GBM cell proliferation over time. Data are presented as mean ± SD. *P < 0.05 and **P < 0.01 vs. NC; n = 3 biological replicates.

To clarify the functional relevance of ARSD in GBM cells, we overexpressed or knocked down ARSD in three GBM cell lines: U87MG, U251MG, and A172. Quantitative real-time PCR showed that ARSD mRNA levels increased in the overexpression group (OE group) and decreased in the knockdown group (KD group) compared with the negative control group (NC group) in all three cell lines (**Figure 2A**). Western blot analysis showed corresponding changes in ARSD protein levels after ARSD overexpression or knockdown (**Figure 2B**).

Next, we examined whether ARSD affects three-dimensional spheroid growth. In spheroid culture, ARSD overexpression increased spheroid size and volume in U87MG, U251MG, and A172 cells compared with the NC group, whereas ARSD knockdown inhibited spheroid growth (**Figure 2C**). Annexin V/PI flow cytometry showed that ARSD overexpression reduced the apoptotic fraction in all three GBM cell lines, while ARSD knockdown increased apoptosis (**Figure 2D**). In addition, the CCK-8 assay showed that ARSD overexpression enhanced GBM cell proliferation over time, whereas ARSD knockdown inhibited proliferative capacity (**Figure 2E**). These results indicate that ARSD contributes to GBM growth-associated malignant phenotypes by promoting proliferation and spheroid formation and reducing apoptosis.

### 3.3 ARSD promotes invasion and matrigel-based tube-like network formation in GBM cells

**Figure 3:**
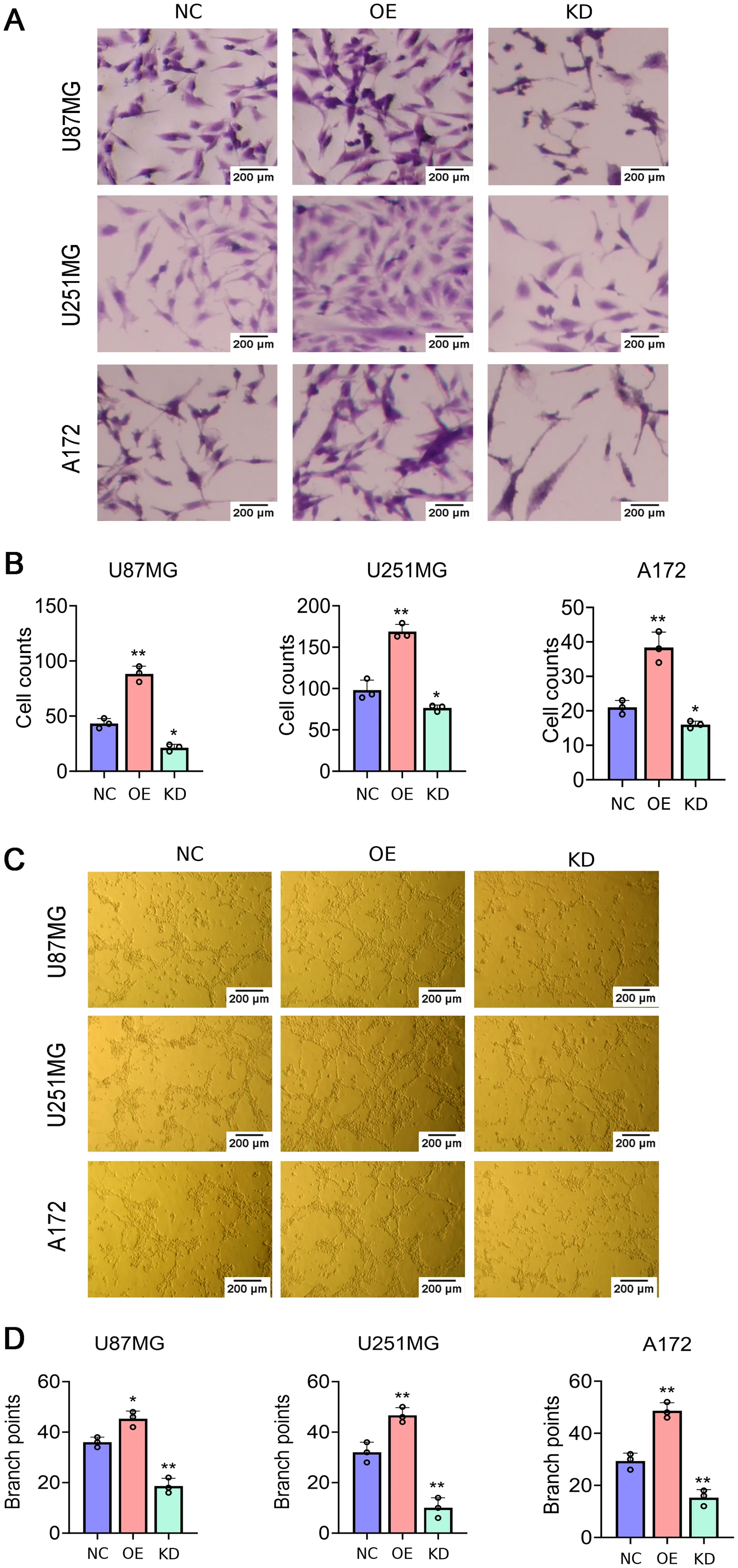
ARSD promotes invasion and Matrigel-based tube-like network formation in GBM cells. ARSD overexpression (OE) and knockdown (KD) were performed in U87MG, U251MG, and A172 cells. **(A)** Representative images of Matrigel-coated Transwell invasion assays. Scale bars: 200 μm. **(B)** Quantification of invaded cells in the Transwell invasion assay. **(C)** Representative images of Matrigel-based tube-like network formation assays. Scale bars: 200 μm. **(D)** Quantification of branch points reflecting tube-like network formation. Data are presented as mean ± SD. *P < 0.05 and **P < 0.01 vs. NC; n = 3 biological replicates.

We next examined whether ARSD affects the invasive capacity of GBM cells. Matrigel-coated Transwell invasion assays showed that ARSD overexpression increased the number of invaded U87MG, U251MG, and A172 cells compared with the NC group, whereas ARSD knockdown reduced the number of invaded cells (**Figures 3A, B**). These results indicate that ARSD promotes the invasive phenotype of GBM cells.

We further assessed the effect of ARSD on short-term tube-like network formation on Matrigel. In Matrigel-based tube formation assays, ARSD overexpression increased tube-like network formation in all three GBM cell lines, as reflected by increased branch-point numbers. Conversely, ARSD knockdown weakened tube-like structure formation relative to the NC group (**Figures 3C, D**). Together, these findings indicate that ARSD promotes GBM cell invasion and short-term tube-like network formation on Matrigel.

### 3.4 ARSD is associated with altered Merlin/LATS/YAP phosphorylation-related protein patterns in GBM cells

**Figure 4:**
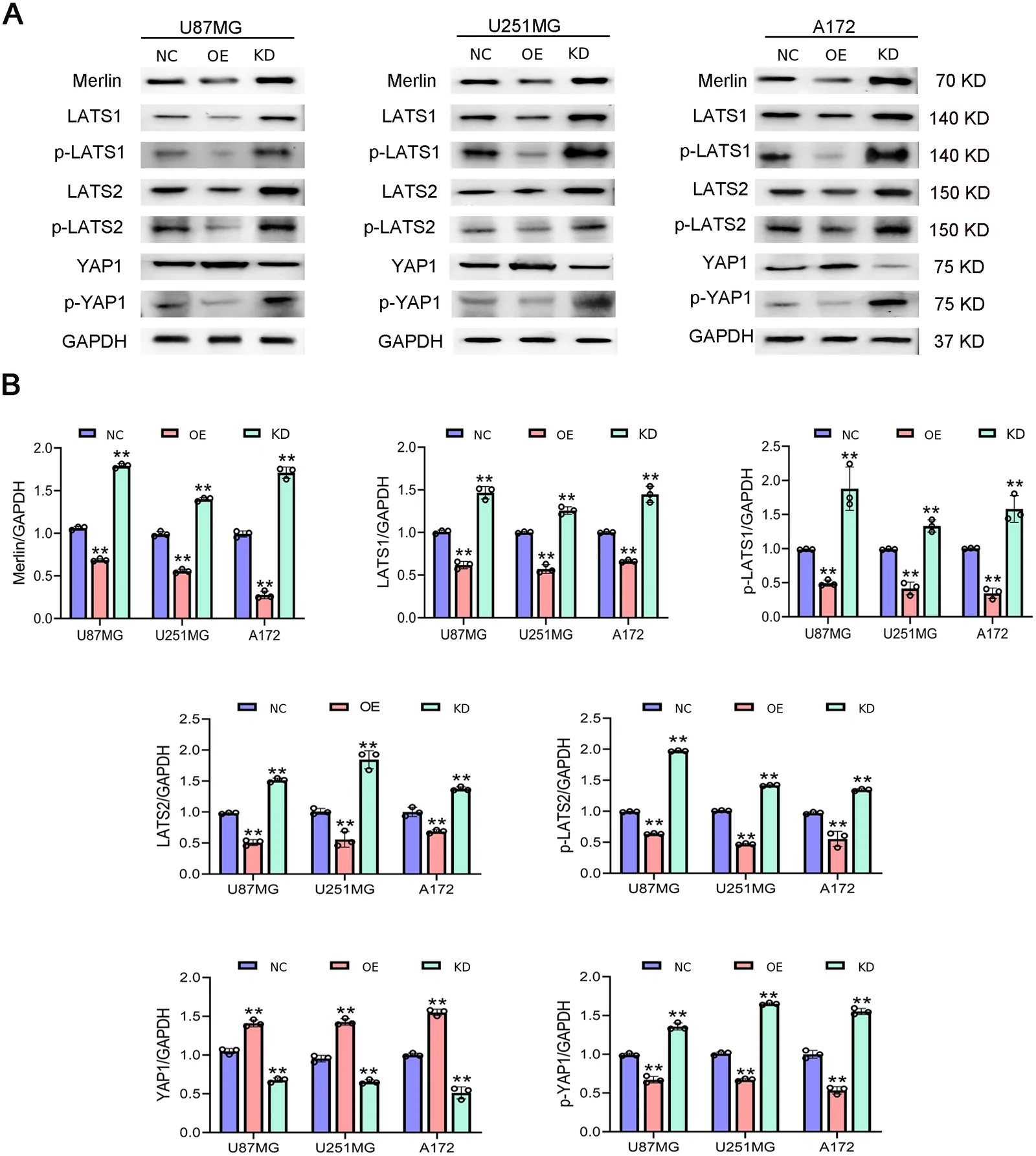
ARSD is associated with altered Merlin/LATS/YAP phosphorylation-related protein patterns in GBM cells. ARSD overexpression (OE) and knockdown (KD) were performed in U87MG, U251MG, and A172 cells. **(A)** Western blot analysis of ARSD-associated changes in Merlin/LATS/YAP phosphorylation-related proteins. **(B)** Quantification of Western blot results. Total Merlin, LATS1, LATS2, and YAP1 protein levels were normalized to GAPDH, whereas phosphorylated LATS1, phosphorylated LATS2, and phosphorylated YAP1 were normalized to their corresponding total protein levels. Data are presented as mean ± SD. *P < 0.05 and **P < 0.01 vs. NC; n = 3 biological replicates.

Because the Merlin/LATS/YAP axis is closely related to tumor cell proliferation, apoptosis, and malignant progression, we next examined whether ARSD expression was associated with changes in Merlin/LATS/YAP phosphorylation-related protein patterns in GBM cells. Western blot analysis showed that ARSD overexpression reduced Merlin, LATS1, and LATS2 protein levels, whereas ARSD knockdown increased the levels of these proteins in U87MG, U251MG, and A172 cells (**Figures 4A, B**). In contrast, total YAP1 was elevated in ARSD-overexpressing cells but reduced in ARSD-knockdown cells (**Figures 4A, B**).

We further quantified the phosphorylation ratios of LATS1, LATS2, and YAP1 by normalizing phosphorylated proteins to their corresponding total protein levels. ARSD overexpression generally reduced the p-YAP1/YAP1 ratio, whereas ARSD knockdown increased p-YAP1/YAP1 in all three GBM cell lines, although the change in the A172 OE group did not reach statistical significance (**Figures 4A, B**). The phosphorylation ratios of LATS1 and LATS2 showed more cell line-dependent changes, with significant differences observed only in selected cell lines and treatment groups (**Figures 4A, B**). These findings indicate that ARSD expression is linked to altered Merlin/LATS/YAP phosphorylation-related protein patterns, especially YAP1 abundance and YAP1 phosphorylation status, in GBM cells.

### 3.5 Irosustat treatment alters ARSD/YAP-associated protein patterns and increases apoptosis in xenograft tumors

**Figure 5:**
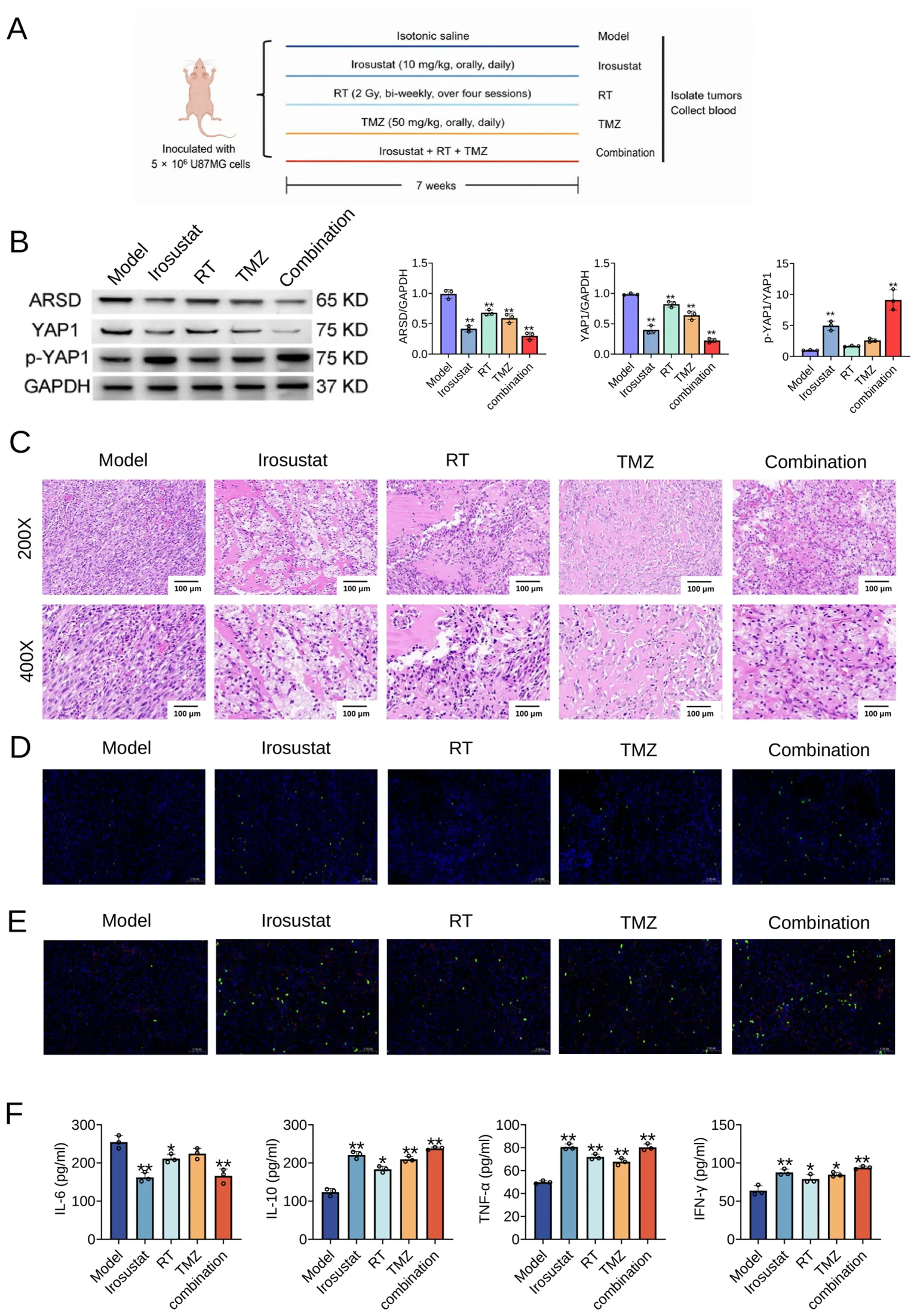
Irosustat treatment alters ARSD/YAP-associated protein patterns and increases apoptosis in xenograft tumors. **(A)** Schematic illustration of the *in vivo* treatment schedule in the subcutaneous U87MG xenograft model. Mice were assigned to the Model, Irosustat, radiotherapy (RT), temozolomide (TMZ), and Combination groups. Tumor tissues and blood samples were collected after 7 weeks of treatment. **(B)** Western blot analysis and quantification of ARSD, YAP1, and phosphorylated YAP1 in xenograft tumor tissues. ARSD and total YAP1 were normalized to GAPDH, and phosphorylated YAP1 was normalized to total YAP1. **(C)** Representative hematoxylin and eosin (H&E) staining of xenograft tumor tissues. Scale bars: 100 μm. **(D)** Representative TUNEL staining of xenograft tumor tissues. Scale bars: 100 μm. **(E)** Representative immunofluorescence staining showing CD68- and CD86-associated signals in xenograft tumor tissues. Scale bars: 100 μm. **(F)** ELISA analysis of circulating IL-6, IL-10, TNF-α, and IFN-γ levels in mouse plasma after treatment. Data are presented as mean ± SD. *P < 0.05 and **P < 0.01 vs. Model; For the histological, immunofluorescence, and ELISA analyses, field-level or technical replicate measurements were averaged to obtain one value per mouse; n = 3 mice per group.

To investigate the *in vivo* relevance of pharmacological sulfatase inhibition in GBM, we established a subcutaneous U87MG xenograft model in immunodeficient mice. When tumors reached the indicated size, mice were randomly assigned to five groups: Model, Irosustat, radiotherapy (RT), temozolomide (TMZ), and Combination treatment with Irosustat, RT, and TMZ (**Figure 5A**).

Western blot analysis of xenograft tumors showed that ARSD protein levels decreased after Irosustat treatment compared with the Model group, with a similar decrease in the Combination group (**Figure 5B**). RT and TMZ alone also had moderate effects on ARSD expression. Total YAP1 protein levels decreased after Irosustat-containing treatment, whereas the p-YAP1/YAP1 ratio increased, particularly in the Irosustat and Combination groups (**Figure 5B**). These results indicate that Irosustat treatment is associated with changes in ARSD/YAP-related protein patterns in xenograft tumors.

Histological examination by H&E staining showed high tumor cell density in the Model group, whereas tumors from treatment groups, especially the Irosustat and Combination groups, showed evident histological alterations and reduced tumor cell density (**Figure 5C**). TUNEL staining further showed enhanced apoptotic signals in xenograft tumor sections after Irosustat, TMZ, and Combination treatment, indicating increased tumor cell apoptosis *in vivo* (**Figure 5D**).

We also examined immune-related markers in tumor tissues by immunofluorescence staining. Compared with the Model group, CD68^-^ and CD86^-^associated signals were increased in tumors from several treatment groups, indicating treatment-associated changes in the measured macrophage-related markers. These findings do not establish macrophage repolarization. (**Figure 5E**). Peripheral blood ELISA analysis also showed treatment-related cytokine changes, including decreased IL-6 levels and increased IL-10, TNF-α, and IFN-γ levels (**Figure 5F**). Together, these data indicate treatment-associated changes in ARSD/YAP-related protein patterns, tumor-cell apoptosis, macrophage-associated markers, and circulating cytokines in U87MG xenograft-bearing mice.

### 3.6 Effects of Irosustat monotherapy and the three-agent combination regimen in a subcutaneous U87MG xenograft model

**Figure 6:**
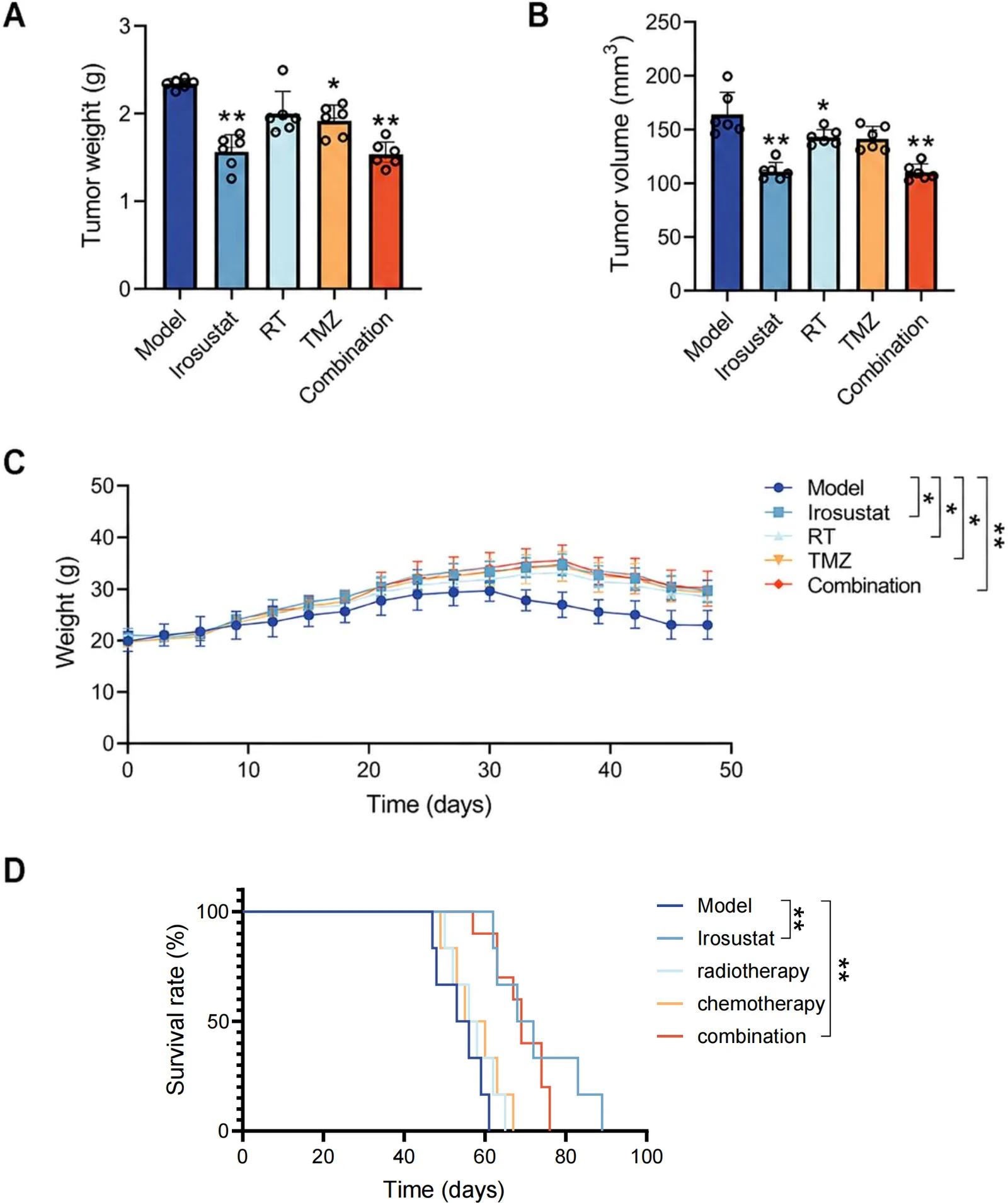
Effects of Irosustat monotherapy and the three-agent combination regimen in a subcutaneous U87MG xenograft model. **(A)** Quantification of tumor weight in the Model, Irosustat, RT, TMZ, and Combination groups. **(B)** Quantification of tumor volume in different treatment groups at the end of treatment. **(C)** Body weight changes of mice during the treatment period. **(D)** Kaplan–Meier survival curves of tumor-bearing mice in different treatment groups. Data are presented as mean ± SD where applicable. *P < 0.05 and **P < 0.01 vs. Model; n = 6 mice per group.

We next assessed how Irosustat-based treatment influenced tumor growth in the subcutaneous U87MG xenograft model. At the treatment endpoint, tumor weight was lower in the Irosustat, TMZ, and Combination groups than in the Model group, whereas the RT group showed a moderate downward trend (**Figure 6A**). Consistently, tumor volume was decreased after Irosustat, RT, and Combination treatment compared with the Model group, with the Combination group showing a marked inhibitory effect on tumor burden (**Figure 6B**).

Mouse body weight was monitored throughout the treatment period to assess general condition and tolerability. No obvious treatment-associated body weight loss was observed in the Irosustat, RT, TMZ, or Combination groups during the observation period (**Figure 6C**). Kaplan-Meier analysis further showed that Irosustat and Combination treatment extended the survival of tumor-bearing mice relative to the Model group (**Figure 6D**). Overall, Irosustat monotherapy was associated with reduced xenograft tumor burden and prolonged survival relative to the Model group. The three-agent Combination group also showed reduced tumor burden and prolonged survival. However, because the study did not include all pairwise treatment combinations or a formal interaction analysis, these results do not establish additive or synergistic interactions among Irosustat, RT, and TMZ.

## 4 Discussion

GBM remains a highly aggressive brain tumor marked by diffuse invasion, therapeutic resistance, marked heterogeneity, and frequent recurrence. Identifying molecules that contribute to GBM malignant phenotypes is therefore important for understanding disease progression and developing new therapeutic strategies. In this study, ARSD was found to be elevated in GBM tissues, and higher ARSD expression correlated with worse overall survival. Functional assays showed that ARSD overexpression enhanced GBM cell proliferation, three-dimensional spheroid growth, invasion, and vasculogenic mimicry-like tube formation, while reducing apoptosis. Conversely, ARSD knockdown suppressed these malignant phenotypes. These findings suggest that ARSD acts as a functional regulator associated with GBM progression.

The use of three-dimensional spheroid culture provides additional support for the biological relevance of our *in vitro* findings. Compared with conventional two-dimensional monolayer culture, 3D spheroid models better reflect several aspects of tumor architecture, cell–cell interaction, extracellular matrix-related constraints, and heterogeneous treatment response (27, 28). In our study, ARSD overexpression increased spheroid growth in U87MG, U251MG, and A172 cells, whereas ARSD knockdown reduced spheroid size and volume. These results indicate that ARSD supports GBM cell growth not only in standard proliferation assays but also under a more spatially organized culture condition. Nevertheless, spheroid models remain simplified systems and cannot fully reproduce the brain tumor microenvironment, vascular niche, immune components, or blood–brain barrier.

ARSD also promoted invasion and short-term tube-like network formation on Matrigel in GBM cells. Invasion is a defining biological feature of GBM and contributes to incomplete tumor resection and recurrence. Our Matrigel-coated Transwell assays showed that ARSD overexpression enhanced invasive capacity, whereas ARSD knockdown reduced invasion. ARSD overexpression also increased tube-like network formation on Matrigel, whereas ARSD knockdown suppressed this phenotype. Vasculogenic mimicry has been reported in GBM and is considered a tumor cell-associated vascular-like phenotype distinct from classical endothelial-cell-driven angiogenesis (15, 16, 29). However, the present 6-h Matrigel assay primarily measures cell alignment and short-term network formation under *in vitro* conditions. Because endothelial-marker co-localization and perfusion-based *in vivo* validation were not performed, the observed networks should not be interpreted as definitive evidence of bona fide vasculogenic mimicry or functional vascular channels.

At the molecular level, ARSD expression was associated with altered Merlin/LATS/YAP phosphorylation-related protein patterns. The Hippo/YAP axis regulates cell proliferation, survival, plasticity, and therapy resistance, and YAP/TAZ activity has been implicated in glioma progression and GBM stemness (9–14, 30–32). In the present study, ARSD overexpression reduced Merlin, LATS1, and LATS2 protein levels and increased total YAP1, whereas ARSD knockdown showed the opposite pattern. ARSD overexpression generally reduced the p-YAP1/YAP1 ratio, while ARSD knockdown increased the p-YAP1/YAP1 ratio. In contrast, the p-LATS1/LATS1 and p-LATS2/LATS2 ratios showed more cell-line-dependent changes. These findings support an association between ARSD expression and Merlin/LATS/YAP-related protein changes, particularly YAP1 abundance and phosphorylation status. However, the present study did not determine whether YAP signaling is required for ARSD-mediated malignant phenotypes. Therefore, these findings should not be interpreted as evidence of a complete causal Hippo/YAP signaling cascade. Future studies incorporating YAP gain- and loss-of-function rescue experiments, YAP subcellular localization analysis, TEAD reporter assays, and downstream target assessment are warranted.

The context-dependent role of ARSD should also be considered. A recent study reported that ARSD promoted glioma progression through the JAK2/STAT3 pathway and macrophage-associated immune infiltration (17), supporting a tumor-promoting function of ARSD in glioma. Conversely, in triple-negative breast cancer cells, ARSD was reported to reduce proliferation and migration by activating Hippo/YAP signaling (18). These differences suggest that ARSD may exert tumor-type-specific effects depending on cellular context, lineage-specific transcriptional programs, and pathway crosstalk. More broadly, recent evidence suggests that epigenetic and transcriptional dysregulation can converge on downstream effectors that promote malignant and metastatic phenotypes in cancer (33). Although the tumor type and upstream regulators differ from those examined in the present study, these findings raise the possibility that ARSD-associated signaling may interact with epigenetic remodeling and Hippo/YAP-related programs in GBM. This possibility remains speculative and warrants further investigation (33). The precise mechanism linking a lysosomal sulfatase such as ARSD to Merlin/LATS/YAP-associated protein changes remains unclear and warrants further investigation.

*In vivo*, Irosustat was used to explore whether pharmacological sulfatase inhibition could affect GBM xenograft growth. Irosustat is an orally active irreversible steroid sulfatase inhibitor that has been evaluated in preclinical and clinical studies (19, 20, 34–36). In our subcutaneous U87MG xenograft model, Irosustat monotherapy was associated with reduced ARSD protein levels, altered YAP phosphorylation-related protein patterns, increased tumor cell apoptosis, reduced tumor burden, and prolonged survival. These findings provide preliminary evidence that pharmacological sulfatase inhibition may influence GBM xenograft growth. Importantly, Irosustat is not an ARSD-specific inhibitor; therefore, the *in vivo* findings should not be interpreted as direct evidence that selective ARSD inhibition alone accounts for the observed antitumor effects. The three-agent Combination group simultaneously received Irosustat, RT, and TMZ. Because the study did not include all pairwise treatment combinations or a formal interaction analysis, the contribution of each treatment component cannot be separated, and the results should not be interpreted as evidence of additive or synergistic interactions.

Several limitations should be acknowledged. First, additional rescue experiments are required to determine whether YAP-related changes are necessary for ARSD-mediated phenotypes. Second, only male mice were used, and sex-related effects were not assessed. Third, the subcutaneous U87MG xenograft model does not reproduce the intracranial microenvironment, blood–brain barrier, brain-specific drug distribution, or the complete immune context of GBM. Accordingly, the CD68/CD86 and cytokine findings remain exploratory and do not establish macrophage repolarization or restoration of antitumor immunity (37–39). Finally, the RT and TMZ schedules were preclinical regimens rather than equivalents of the clinical Stupp protocol, and further studies using more selective genetic and pharmacological approaches are warranted.

In short, our results show that the expression of ARSD in GBM is elevated and is associated with poor prognosis. ARSD promotes the proliferation of GBM cells, globular growth, invasion and the formation of angiogenesis mimic tubular structures, while inhibiting cell apoptosis. In conclusion, ARSD is upregulated in GBM and is associated with unfavorable patient survival. ARSD overexpression promotes GBM cell proliferation, spheroid growth, invasion, and Matrigel-based tube-like network formation while reducing apoptosis, whereas ARSD knockdown produces the opposite effects. ARSD expression is associated with altered Merlin/LATS/YAP phosphorylation-related protein patterns, particularly changes in YAP1 abundance and phosphorylation status. These findings do not establish that YAP signaling is required for ARSD-mediated phenotypes, and further mechanistic validation is warranted. In the subcutaneous U87MG xenograft model, Irosustat monotherapy was associated with reduced tumor burden and prolonged survival. The three-agent combination regimen also showed antitumor activity; however, because all pairwise treatment combinations and formal interaction analyses were not included, additive or synergistic effects were not evaluated.

## Data Availability

The datasets presented in this study can be found in online repositories. The names of the repository/repositories and accession number(s) can be found in the article/**Supplementary Material**.
